# Genomewide Pattern of Synonymous Nucleotide Substitution in Two Complete Genomes of *Mycobacterium tuberculosis*

**DOI:** 10.3201/eid0811.020064

**Published:** 2002-11

**Authors:** Austin L. Hughes, Robert Friedman, Megan Murray

**Affiliations:** * University of South Carolina, Columbia, South Carolina, USA; † Harvard School of Public Health, Boston, Massachusetts, USA

**Keywords:** genome evolution, Mycobacterium tuberculosis, nucleotide diversity, synonymous substitution

## Abstract

Comparison of the pattern of synonymous nucleotide substitution between two complete genomes of Mycobacterium tuberculosis at 3,298 putatively orthologous loci showed a mean percent difference per synonymous site of 0.000328 ± 0.000022. Although 80.5% of loci showed no synonymous or nonsynonymous nucleotide differences, the level of polymorphism observed at other loci was greater than suggested by previous studies of a small number of loci. This level of nucleotide difference leads to the conservative estimate that the common ancestor of these two genotypes occurred approximately 35,000 ago, which is twice as high as some recent estimates of the time of origin of this species. Our results suggest that a large number of loci should be examined for an accurate assessment of the level of nucleotide diversity in natural populations of pathogenic microorganisms.

Surveys of genetic diversity in the pathogenic bacterium Mycobacterium tuberculosis have revealed a contradictory picture. In spite of known polymorphism at the phenotypic level and abundant polymorphism associated with repetitive elements (1), surveys of single nucleotide polymorphism in protein-coding genes have shown surprisingly low levels of polymorphism in comparison with other eubacterial species (2). The apparent low level of nucleotide polymorphism has led to the hypothesis that the ancestor of this species occurred quite recently, perhaps 15,000–20,000 years ago (2,3). However, if the number of substitutions per site is low, the error of estimation of this number would be expected to be substantial unless a very large number of sites are surveyed. We addressed the question of polymorphism in M. tuberculosis by comparing protein-coding genes in two completely sequenced genotypes, H37Rv and CDC1551 (4).

## Methods

We applied the BLASTP program (5) to identify, for each predicted protein sequence in the H37Rv genome (GenBank accession no. AL123456), the closest homolog in the CDC1551 genome (GenBank accession no. AE000516). Following GenBank annotations, we compared 3,972 predicted proteins in H37Rv with 4,187 predicted proteins in CDC1551. We used a strict search criterion (E = 10-50) to identify truly orthologous gene pairs. We aligned (6) the putative orthologous pairs of amino acid sequences (N = 3,428), then imposed this alignment on the DNA sequences.

Visual inspection of amino acid alignments showed that certain alignments, usually near the N-terminus or C-terminus, had regions of very low sequence identity. Examination of the DNA sequences of the corresponding genes showed that these regions of low identity were typically caused by a frameshift in one of the two genomes relative to the other. Whether these frameshifts are biologically real or result from sequencing error was uncertain; therefore, we eliminated 119 such gene pairs from our data set. For the remaining gene pairs (N = 3,309), we computed the proportion of synonymous substitutions per synonymous site (pS) and the proportion of nonsynonymous substitutions per site (pN) by using Nei and Gojobori’s method (7). Because values of pS and pN were very low in most cases, we did not correct for multiple hits.

Because pS values appeared to fall into two groups (see Results), we used a simple probabilistic model to separate these two sets of gene pairs. We assumed that the probability of synonymous substitution followed two separate binomial distributions, designated models A and B, with probabilities of “success” (i.e., of a synonymous difference) designated pA and pB, respectively. Using the Bayes equation, for each gene pair with a given pS value, we computed the probability that model A applies, given the observed pS:P(A|pS) = (pSA) fA /[(pSA) fA + (pSB) fB],

where fA is the frequency of cases to which model A applies, fB the frequency of cases to which model B applies, pSA is the binomial probability of obtaining the observed pS, given the number of synonymous sites in the gene and a probability of a synonymous difference equal to pA; and pSB is the binomial probability of obtaining the observed pS, given the number of synonymous sites in the gene and a probability of a synonymous difference equal to pB. The probability that model B applies, given pS, is

## Results

Of 3,309 pairs of putatively homologous protein-coding genes in the H37Rv and CDC1551 genomes of M. tuberculosis, 2,662 (80.5%) showed no synonymous or nonsynonymous nucleotide differences between the two genomes, and 3,010 (91.0%) showed no synonymous differences between the two genomes. However, in a small number of gene pairs, the proportion of synonymous differences per synonymous site (pS) was surprisingly high. In 13 (0.4%) gene pairs, pS was >0.01, and in 3 gene pairs pS exceeded 4%. These extreme pS values seen in a small number of gene pairs are much higher than generally observed between alleles at neutrally evolving loci in eukaryotes (8). Thus, the comparison of protein-coding genes between the two M. tuberculosis genomes suggested the existence of two distinct groups of gene pairs: a large group having few or no synonymous differences and a much smaller group with a substantial degree of synonymous divergence.

We used a simple probabilistic model (see Methods) to separate these two sets of gene pairs, designated Group A and Group B, respectively (Figure). The application of this method showed 11 loci with unusually high *p_S_* values and probabilities of assignment to group A of <50% (Figure).

**Figure:**
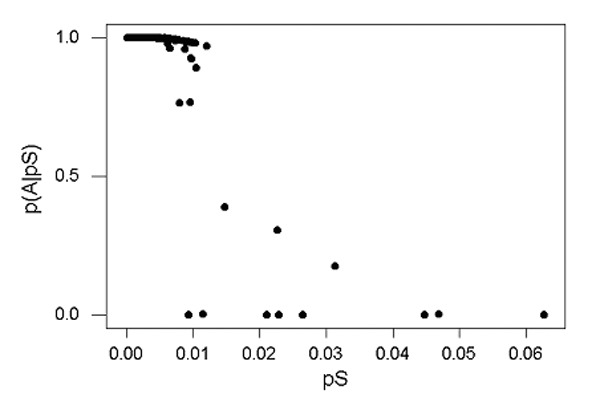
A plot of p(A|*p_S_*), the probability of assignment of a locus to group A given the observed *p_S_* value, as a function of *p_S_* at 3,309 loci compared between the H37Rv and CDC1551 genotypes of *Mycobacterium tuberculosis*. The plot shows the bimodal nature of the distribution of *p_S_* values, with overall higher values of *p_S_* at the 11 loci having p(A|*p_S_*) >50%.

We assumed that Group A members are truly orthologous gene pairs that diverged at the time of the common ancestor of the H37Rv and CDC1551 genomes. Group A included 3,298 pairs, with mean pS for all genes of 0.000328 ± 0.000022 standard error. When pS was estimated for the 3,298 genes concatenated together (a total of 934,413 synonymous sites), an estimate of pS = 0.000348 ± 0.00019 was obtained. The range of pS values in Group A was between zero and 0.012; a total of 288 loci in Group A had pS values other than zero. These results show a substantial level of nucleotide diversity, approximately half the level of nucleotide diversity in humans (9).

Rates of nucleotide substitution per unit time are difficult to estimate in bacteria given the lack of calibration from the fossil record (10). To obtain an estimate of the rate of synonymous nucleotide substitution, we used published data on comparisons of Escherichia coli and Salmonella typhimurium (11,12), which are believed to have diverged approximately 100 million years ago (13,14) (Table 1). This procedure yielded estimates for the last common ancestor of H37Rv and CDC1551 in the range of 34,000–38,000 years (Table 1). These estimates are approximately twice previous estimates of the age of the common ancestor of worldwide M. tuberculosis (2,3). To obtain the observed mean pS value between H37Rv and CDC1551 within 15,000–20,000 years would require a rate of synonymous substitution approximately twice that observed in Enterobacteria.

**Table 1:** Estimates^a^ of the divergence time of the H37Rv and CDC1551 genotypes of *Mycobacterium tuberculosis*

Reference	No. loci	Synomymous substitutions/site/yr	Divergence time (H37Rv and CDC1551)
11	67	4.7 ± 0.2 X 10^-9^	34,900 ± 2,300 ^b^ (33,500–36,400) ^c^
12	128	4.4 X 10^-9^	37,300 ± 2,500 ^b^

^a^Based on synonymous substitutions between *Escherichia coli* and *Salmonella typhimurium*, assumed to have diverged 100 million years ago (13,14). ^b^Estimates are shown ± standard error, based on standard error of mean *p_S_*. ^c^Range based on standard error of rate estimate.

Group B consisted of 11 gene pairs with mean pS of 0.0286 ± 0.0050 (Table 2).

**Table 2:** Proteins for which the nearest homologous comparison between the H37Rv and CDC1551 genotypes of *Mycobacterium tuberculosis* has a high *p_S_* value (Group B)

	Accession nos.	Protein function	*p_S_*	*p_N_*
Probable differential deletion				
	NP_216309, NP_335079	unknown	0.0470	0.0000
	NP_215713, NP_335504	unknown	0.0628	0.0043
	NP_216319, NP_336310	PE repeat family	0.0115	0.0094
	NP_215965, NP_335949	PE repeat family	0.0448	0.0185
Possible horizontal gene transfer				
	NP_214910, NP_334815	unknown	0.0226	0.0105
	NP_216104, NP_336077	unknown	0.0265	0.0084
	NP_216281, NP_336535	unknown	0.0210	0.0161
	NP_215835, NP_335809	adenylate cyclase	0.0229	0.0068
	NP_216564, NP_336573	polyketide synthase	0.0093	0.0036
	NP_217029, NP_337080	unknown	0.0148	0.0156
	NP_216862, NP_335679	unknown	0.0313	0.0000
Mean ± S.E.			0.0286 ± 0.0050	0.0085 ± 0.0019^a^

^a^Paired sample t-test of the hypothesis that *p_S_* = *p_N_* , p<0.01. The quantities *p_S_* and *p_N_* are the proportion of nucleotide difference per synonymous site and per nonsynonymous site, respectively.

In Enterobacteria, a negative correlation exists between observed proportions of synonymous difference and codon bias (11). In the case of Mycobacterium, codon bias results mainly from the very high third position G+C content of most genes (15). In our data, however, we observed no correlation between pS and proportion G+C at third codon positions (r = -0.010; not significant).

## Discussion

A number of additional possibilities may explain the occurrence of gene pairs with higher than expected pS values: 1) Balanced polymorphism. Selectively maintained polymorphisms are expected to be much older than neutral polymorphisms and may even predate speciation events (16). In the case of haploid organisms such as bacteria, balancing selection would take the form of frequency-dependent selection rather than overdominant selection. 2) Differential deletion. In a multi-gene family, if one member of an orthologous pair of genes were deleted in one genotype, the gene pairs would involve paralogous, not orthologous comparisons. 3) Horizontal gene transfer. A gene obtained by one of the two genotypes from another bacterial species would be expected to be more divergent than other genes in that genotype.

One indication of a balanced polymorphism is a higher rate of nonsynonymous than synonymous substitution (8). There was no strong evidence of such selection in the present case; pS was greater than pN at 10 of the 11 loci, and pN exceeded pS only slightly at one locus (Table 2). In addition, we compared pS and pN in sliding windows of 30 codons along the length of these genes. No regions were observed in which pN was greater than pS (data not shown). Thus, there was no evidence of positive selection acting on specific regions of these genes. On the other hand, differential deletion can probably explain some cases, most notably members of the PE multi-gene family (11) (Table 2). The remaining gene pairs are possibly cases of horizontal gene transfer (Table 2), for which there is some recent evidence in M. tuberculosis (17). Presumably a related species of Mycobacterium was the source of such gene transfers.

Our results did not support the hypothesis that the common ancestor of M. tuberculosis was relatively recent (2). Rather, the pattern of nucleotide substitution at synonymous sites suggested a divergence time for the two available genotypes of this species approximately 35,000 years ago. Since H37Rv and CDC1551 represent two genotypes sampled from within the species, they are probably not the most divergent genotypes possible. Thus, the last common ancestor of M. tuberculosis likely occurred considerably earlier than 35,000 years ago.

While the difference between an estimate of 15,000–20,000 years and one of 35,000 years is not large on an evolutionary time scale, such a difference is substantial on the scale of human history. For example, the existence of two genotypes in the current population of M. tuberculosis with a common ancestor at 35,000 years is evidence against the hypothesis that M. tuberculosis arose, presumably from M. bovis, at the time of human domestication of cattle (18). Our result is thus consistent with phylogenetic analyses based on insertion-deletion events, which suggest that the M. tuberculosis lineage was a human pathogen well before the origin of M. bovis (19). Thus, along with recent evidence of an ancient origin and extensive polymorphism in the malaria parasite Plasmodium falciparum (20,21), our study provides evidence against the long-held view that virulent pathogens are invariably evolutionarily recent (22).

Our estimate is conservative because the rate of synonymous substitution may actually be lower in Mycobacterium than in Enterobacteriaceae, given the highly skewed G+C content in the former. Furthermore, our estimate of the mean proportion of synonymous difference was conservative because we excluded 119 loci with potential frameshifts between the two genotypes as well as a set of 12 loci with unusually high pS values. In addition to the 12 loci assigned to our Group B, certain other loci might also have originated from horizontal gene transfer. However, even if horizontal gene transfer has occurred at other loci besides those in Group B, eliminating further loci with relatively high pS values from Group A will not affect the results greatly. For example, if we eliminate the 10 loci with highest pS values from Group A, mean pS will be reduced only to 0.000299, and the estimated age of the common ancestor will be barely affected.

The degree of polymorphism observed in this study is unlikely to have been substantially influenced by sequencing errors. The error rate for finished sequences from the Institute for Genomic Research (where CDC1551 was sequenced) has been independently estimated at <1 in 88,000 bases (23). Assuming a similar error rate for both 4.4 mega-bp M. tuberculosis genomes, we would expect to see approximately 100 differences between them due to sequencing errors. Approximately 21 such differences would be expected in the 938,778 synonymous sites in Group A and Group B genes. In fact, 411 synonymous differences were observed at these sites; thus, even if present, sequencing errors are likely to have made up only a small fraction (approximately 5%) of the total synonymous polymorphism. At such a rate, sequencing errors would have little effect on our estimates of nucleotide diversity at synonymous sites or the age of the common ancestor of the two genomes.

In addition, the hypothesis that the single nucleotide polymorphisms (SNPs) observed between these genotypes are real received strong support from a recent study that observed a number of the same SNPs in clinical isolates (24). Moreover, since sequencing errors are expected to occur at random with respect to the reading frame of coding sequences, the fact that mean pS exceeded mean pN in both Group A and Group B was strong evidence against the hypothesis that a substantial proportion of the observed polymorphism was due to sequencing error.

Simple considerations of probability can explain why earlier studies produced relatively low estimates of this species’ age. If we assume that the per-site probability of a synonymous difference between two M. tuberculosis genomes is equal to the mean pS observed between H37Rv and CDC1551 (0.000328), then the probability is approximately 95% that no synonymous differences will be seen in a gene with 150 synonymous sites. The probability that no synonymous differences will be seen in 10 such loci chosen at random is approximately 60%, and the probability that no synonymous differences will be observed at 20 such loci is approximately 37%. On the other hand, the probability that no synonymous differences will be seen at 100 such loci is <1%.

These calculations emphasize the need to examine a very large number of nucleotide sites to obtain a reliable estimate of nucleotide diversity and thus of the age of the most recent common ancestor in cases where the frequency of substitution is less than one in a thousand. Even when the frequency of substitution is between one in a thousand and one in a hundred, substantial stochastic error is possible if the number of loci examined is small. Thus, any study that estimates population parameters from nucleotide sequence data needs to survey a substantial number of loci. These considerations are particularly important in the case of pathogenic microorganisms, where a number of factors (including both natural selection and horizontal gene transfer) may lead to substantial differences among loci with respect to the level of nucleotide diversity.

Comparison of two complete genomes of M. tuberculosis showed a greater extent of sequence polymorphism than would be expected on the basis of previous studies, in turn suggesting that analysis of additional genomes will likely show further polymorphism. Polymorphism in any species of pathogen may complicate therapeutic strategies because it implies the existence of variation on which selection can act, including selection imposed by human vaccines and pharmacologic agents (20). On the other hand, known polymorphisms may prove useful to investigators in reconstructing the evolutionary relationships among clinical isolates and in providing markers for understanding the genetic basis of complex phenotypic traits.
